# Clinical Treatises on the Pathology and Therapy of Disorders of Metabolism and Nutrition

**Published:** 1903-10

**Authors:** 


CLINICAL TREATISES ON THE PATHOLOGY AND THERAPY OF DISORDERS OF METABOLISM AND NUTRITION.—By Prof. Dr. Carl von Noorden, Physician in Chief to the City Hospital, Frankfurt-am-Main. Authorized American Edition, translated under the direction of Boardman Reed, M. D., Professor of Diseases of the Gastro-Intestinal Tract, Hygiene and Climatology, Department of Medicine, Temple College; Physician to the Samaritan Hospital, Philadelphia.

PART III.—MEMBRANOUS CATARRH OF THE INTESTINES (Colica Mucosa.) By Prof. Dr. Carl von Noorden, with the collaboration of Dr. Carl Dapper. E. B. Treat & Co., New York. 1903. Price 50 cents.

Professor von Noorden's masterly exposition of the subject of "Membranous Catarrh of the Intestines" covers fully the matter of the etrology and pathology, and treatment of this stubborn disease, and should be in the hands of every general practitioner of medicine. R. & L.

